# Strike-slip 23 January 2018 M_W_ 7.9 Gulf of Alaska rare intraplate earthquake: Complex rupture of a fracture zone system

**DOI:** 10.1038/s41598-018-32071-4

**Published:** 2018-09-12

**Authors:** Anne Krabbenhoeft, Roland von Huene, John J. Miller, Dietrich Lange, Felipe Vera

**Affiliations:** 1 GEOMAR Helmholtz Center for Ocean Research Kiel, Wischhofstr. 1-3, 24148 Kiel, Germany; 2 U.S. Geological Survey, Scientist Emeritus, 800 Blossom Hill Road, Los Gatos, CA 95032 USA; 3 U.S. Geological Survey, Scientist Emeritus, Denver Federal Center, Denver, CO 80225 USA; 4 Helmholtz-Zentrum Potsdam, Deutsches GeoForschungsZentrum GFZ, Telegrafenberg 1, 14473 Potsdam, Germany; 5 Freie Universität Berlin, Malteserstr. 74−100, 12249 Berlin, Germany

## Abstract

Large intraplate earthquakes in oceanic lithosphere are rare and usually related to regions of diffuse deformation within the oceanic plate. The 23 January 2018 M_W_ 7.9 strike-slip Gulf of Alaska earthquake ruptured an oceanic fracture zone system offshore Kodiak Island. Bathymetric compilations show a muted topographic expression of the fracture zone due to the thick sediment that covers oceanic basement but the fracture zone system can be identified by offset N-S magnetic anomalies and E-W linear zones in the vertical gravity gradient. Back-projection from global seismic stations reveals that the initial rupture at first propagated from the epicenter to the north, likely rupturing along a weak zone parallel to the ocean crustal fabric. The rupture then changed direction to eastward directed with most energy emitted on Aka fracture zone resulting in an unusual multi-fault earthquake. Similarly, the aftershocks show complex behavior and are related to two different tectonic structures: (1) events along N-S trending oceanic fabric, which ruptured mainly strike-slip and additionally, in normal and oblique slip mechanisms and (2) strike-slip events along E-W oriented fracture zones. To explain the complex faulting behavior we adopt the classical stress and strain partitioning concept and propose a generalized model for large intra-oceanic strike-slip earthquakes of trench-oblique oriented fracture zones/ocean plate fabric near subduction zones. Taking the Kodiak asperity position of 1964 maximum afterslip and outer-rise Coulomb stress distribution into account, we propose that the unusual 2018 Gulf of Alaska moment release was stress transferred to the incoming oceanic plate from co- and post-processes of the nearby great 1964 M_W_ 9.2 megathrust earthquake.

## Introduction

**Figure 1:**
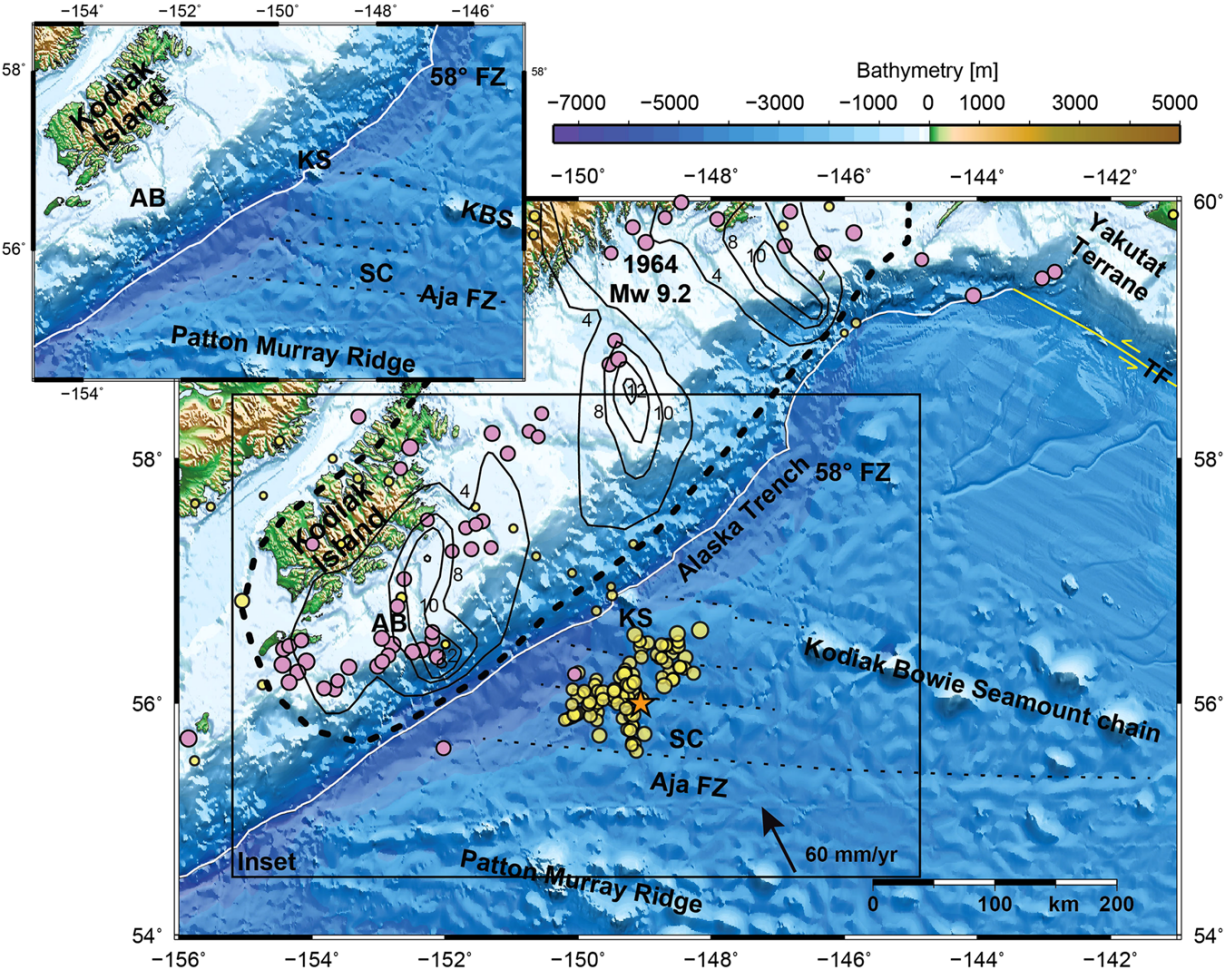
Overview of the Gulf of Alaska shown by GEBCO 2014^28^ bathymetric map. Star marks the 23.01.2018 M_W_ 7.9 Alaska earthquake. Magenta circles mark aftershocks in 1964 and yellow circles mark 2018 aftershocks (2018: M_W_ > 4 on oceanic plate). 1964 events are M_W_ > 5, because of the sparse seismometer recordings (compared with the network available today). Earthquakes are reported in Alaska Earthquake center and US Geological Survey/National Earthquake Information Center (USGS/NEIC) catalog^19^ (https://earthquake.usgs.gov/earthquakes, 2018). Some FZs have bathymetric expression, black dotted lines highlight FZ-associated long wavelength bathymetric minima. Annotated arrow shows plate convergence relative to the North America plate^7^. Bold dashed line shows extent of 1964 M_W_ 9.2 rupture, solid annotated black lines are slip contours of 4, 8, 10 and 12 m, after^17^. Shallow coseismic peak slip of the 1964 M_W_ 9.2 Kodiak asperity rupture^17^ was seaward of Albatross Bank (AB) within a seismic grid. Bathymetric illumination highlights E-W trending structures. Solid white line marks Alaska Trench axis. KS – Kodiak seamount; SC – Surveyor Channel, TF – Transition Fault. **Inset:** Bathymetry is shown without earthquakes and dashed lines highlight FZ-associated long wavelength bathymetric minima. KBS: Kodiak Bowie Seamount chain. Figure made with GMT vers. 4^50^.

**Figure 2:**
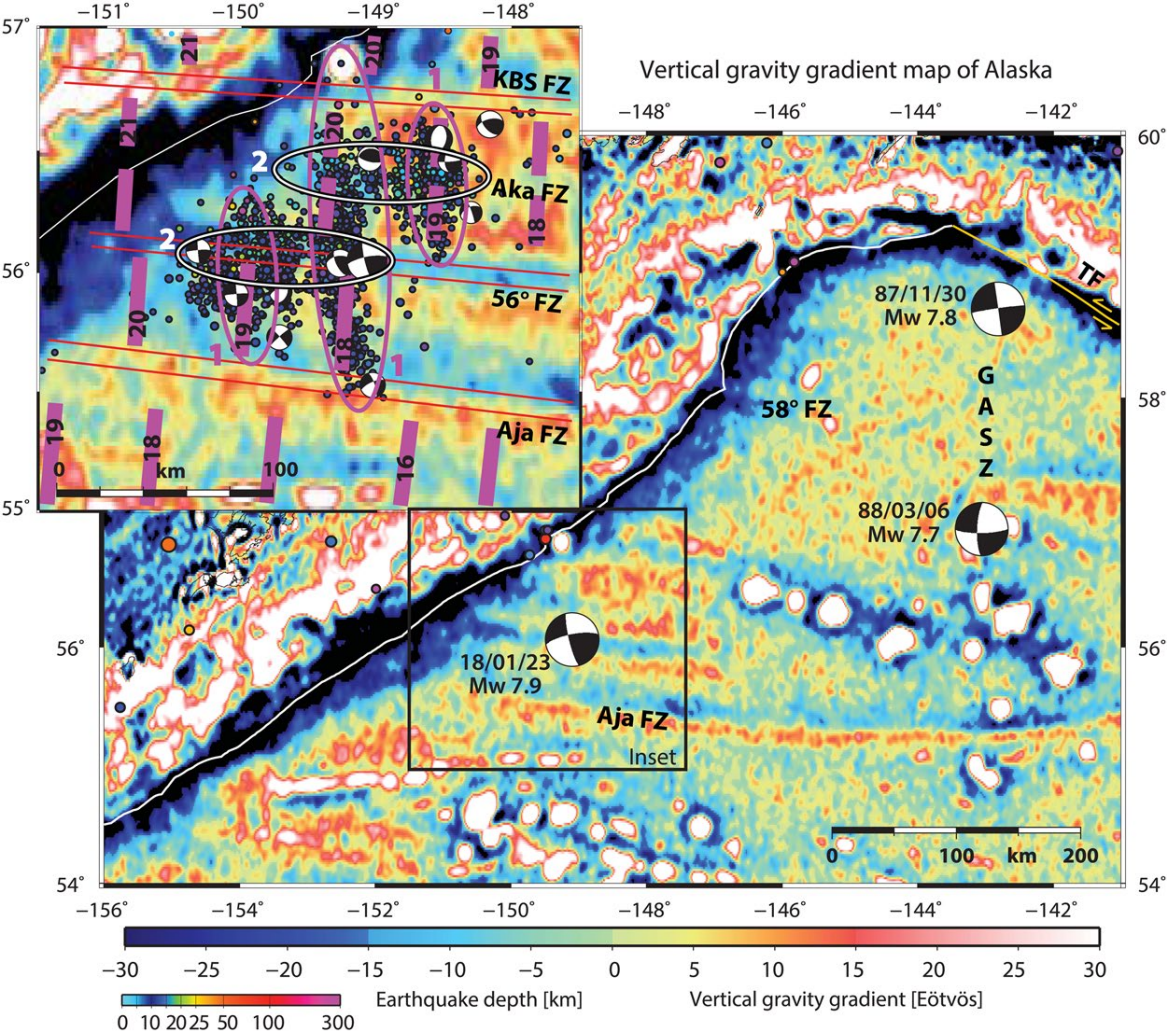
Vertical gravity gradient (VGG; TOPEX^29^) of the Gulf of Alaska. Fracture zones (FZ) are clearly imaged in the VGG. Aftershocks (M_W_ > 3) are plotted from 23.01.2018 to 19.02.2018. Moment tensors from two major Pacific Plate earthquakes: (M_W_ 7.8/7.7 in 1987/1988) ruptured the central Gulf of Alaska Shear Zone (GASZ); and moment tensor of the 23.01.2018 M_W_ 7.9 main shock. **Inset:** Aftershocks of the January 2018 Alaska earthquake and our interpretation of two aftershock groupings marked by ovals: (1) N-S trending parallel to magnetic anomalies, here are two sub-groups with normal and strike-slip mechanisms, (2) E-W trending strike-slip along the Aka and 56°N FZs. Double red lines indicate FZs and annotated magenta lines indicate magnetic anomalies are after^20^. Magnetic anomalies are offset ~85 km at 56°N FZ. Magnetic anomalies and ages at center of aftershock cluster 1: 20 ≈ 43 Ma; 18 ≈ 39 Ma. KBS – Kodiak Bowie Seamount Chain; KS – Kodiak seamount; TF – Transition Fault. Earthquakes and moment tensors are from Alaska Earthquake center and USGS/NEIC catalog^19^. Figure made with GMT vers. 4^50^.

Some of the world’s largest earthquakes occur in Alaska, such as the great 1964 M_W_ 9.2 earthquake (Fig. 1). Most major and great earthquakes are related to rupture of the subduction megathrust. In contrast, many major earthquakes that occur in the oceanic lithosphere near subduction zones are usually bending-related outer-rise normal faulting earthquakes (e.g.^1,2^). Oceanic lithosphere strike-slip earthquakes near subduction zones are rare. Two major Pacific Plate N-S trending strike-slip earthquakes (Fig. 2, upper right; M_W_ 7.8/7.7 in 1987/1988) ruptured a composite length of ~250 km in the central Gulf of Alaska^3^. This region has hosted a spatially persistent cluster of diffuse seismicity since then^4–6^ where complex N-S aftershock patterns dominate and additionally ENE – WSW trending aftershock clusters reactivated fracture zones (FZs)^4^. This cluster of seismicity resulted from a combination of enhanced tensional stress in the Pacific Plate following the 1964 great Alaska earthquake and compressional stress resulting from the collision of the Yakutat Terrane with North America^3,5^. Additionally, the rupture occurred in a zone of weakness in the crust inherited from processes of plate formation^3,5^. Convergence of the Pacific Plate with the North America Plate is currently 60 mm/yr and almost trench-normal (Fig. 1)^7^. However, ocean plate fabric trends N-S and magnetic anomalies are ~30–35° oblique to the Alaska Trench (Fig. 2, inset).

Worldwide, only two other regions have hosted numerous major and great strike-slip ocean lithosphere earthquakes near subduction zones in the documented era: Macquarie Ridge near the Puysegur Trench in the southern Tasman Sea and offshore Sumatra^8^ (and references within). For both regions plate convergence is oblique and the high level of moment release can be explained by diffuse deformation associated with microplates^7^.

A long record of ongoing major and great strike-slip intraplate earthquakes from the southern Tasman Sea documents the complex tectonic setting ~150 km seaward of the obliquely subducting Australia Plate (Puysegur Block) beneath the Pacific Plate where strike-slip motion along the Macquarie Ridge changes to convergence at the Puysegur Trench (e.g.^9^). Several large (M_W_ > 7.5) intra-oceanic plate strike-slip earthquakes (1924, 1926, 1943, 1981, 2004; e.g.^10–12^) intersperse with great interplate strike-slip (e.g. 1989 great M_W_ 8.2 Macquarie Ridge^9,13^) or thrust earthquakes (1979, Puysegur subduction zone^11^). These large earthquakes with different rupture mechanisms show triggering interactions of interplate thrust earthquakes with intraplate strike-slip events on nearby FZs and oceanic fabric, or vice versa^8,14^.

The other region of previous intra-oceanic earthquakes near a subduction megathrust is located offshore northern Sumatra and includes the largest recorded strike-slip intraplate great earthquakes (M_W_ 8.6 & 8.2 of 2012; e.g.^15^) seaward of coseismic peak slip that occurred during the great 2004 Sumatra megathrust earthquake. This earthquake sequence most likely triggered by the M_W_ 9.2 2004 earthquake (e.g.^16^), ruptured a network of en échelon faults orthogonal and parallel to the 90°E Ridge^15^.

The 23 January 2018, 09:31 UTC, M_W_ 7.9 Gulf of Alaska strike-slip earthquake (Fig. 1) is one of the unusual oceanic intraplate earthquakes that rupture areas seaward of a trench with a high potential to produce megathrust earthquakes. In contrast to the other two regions, Alaska is not known for diffuse deformation within the incoming oceanic plate and therefore the moment magnitude of M_W_ 7.9 during the 23 January 2018 Gulf of Alaska main shock leads to the question of how stress accumulated prior to this earthquake. Here, we use the back-projection method to characterize the main shock rupture propagation and bathymetric, seismic, gravity and magnetic field data to identify and characterize the FZ system concealed by thick and deep sea-fan sediment, which ruptured during this unusual type of earthquake.

## Observations

The 2018 M_W_ 7.9 Gulf of Alaska earthquake ruptured the oceanic plate seaward of a peak coseismic slip area of the Kodiak asperity of the great (M_W_ 9.2) 1964 earthquake^17^ (Fig. 1). The epicenter of the 2018 Gulf of Alaska earthquake is located seaward of the marine forearc where the 1964 megathrust slip extended close to the trench axis. The 2018 event is located seaward of a significant group of the 1964 aftershocks and a region of afterslip maximum, indicated by GPS measurements^18^ and additionally, where 1964 outer-rise aftershocks occurred (Fig. 1).

**Figure 3:**
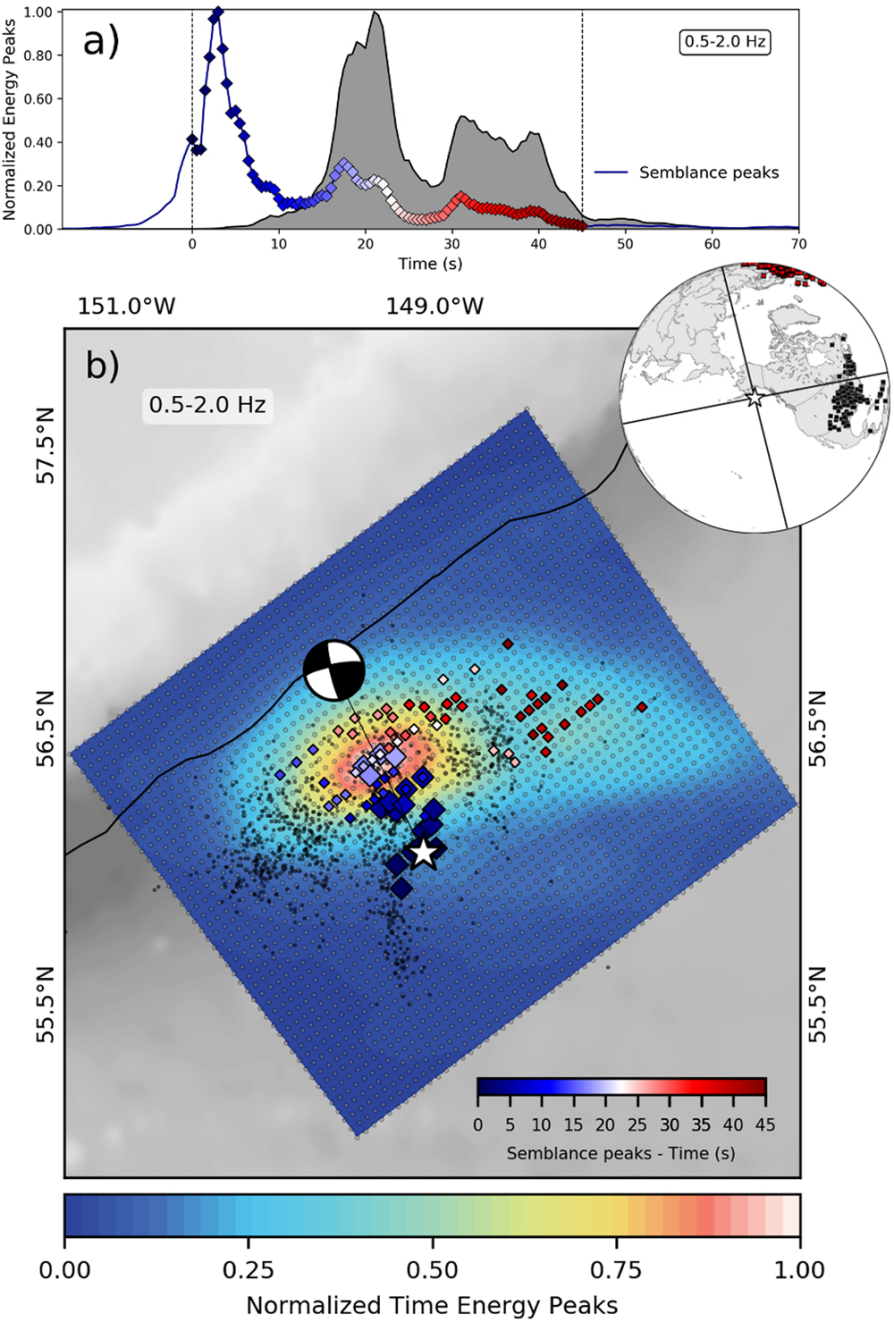
(**a**) Time history of energy (gray shaped area) and semblance peaks (blue curve) centered at time t = 0 by using time windows of L = 6 s. (**b**) Normalized time energy peaks (color image) and semblance peaks (colored diamonds) for the 23 January 2018 M_W_ 7.9 strike-slip Gulf of Alaska earthquake back-projection (0.5–2.0 Hz). The results were constructed by multiplying independent results of both north-central America and Europe waveforms back-projections. White star marks epicenter. Black dots show aftershocks of January 2018 (M ≥ 2.5). Seismicity and focal mechanisms obtained from USGS/NEIC catalog^19^ and subduction margin contour from the slab 1.0 model^51^. **Inset:** station distribution in north-central America (black squares) and Europe (red squares), black lines show the projection of the focal mechanism nodal planes. See also supplementary movie (Movie SM1) showing temporal rupture normalized energy peak propagation. Figure made with Obspy^52^ and matplotlib^53^.

Back-projection reveals that the main shock ruptured an area of ~150 × 60 km (Fig. 3). Initially the rupture started with low energy and propagated from the epicenter^19^ on the 56° FZ to the north along magnetic anomaly 20^20^ (Figs 2 and 3). Rupture propagation most likely was stopped by the Aka FZ (named herein), indicated by the energy emission peak 18–21 s after rupture initiation (Fig. 3; 2^nd^ peak in semblance function). There, the rupture was re-directed and propagated in E-W direction, mainly to the east along the Aka FZ. Such a transfer or termination of great earthquake slip is not commonly reported. After ~50 s the main shock terminated at the easternmost location of the aftershock distribution near ~148°W, on the Aka FZ. For two months after the main shock, aftershocks were distributed along two major tectonic trends (Fig. 2, inset): (1) rupture centered along N-S magnetic anomaly 20 (≈43 Ma)^20^ just seaward of where the Pacific Plate bends into the Alaska Trench and concentrated S-W of the Kodiak Seamount (KS) constriction in the Alaska Trench axis and (2) the E-W oriented FZ system (56°N FZ and Aka FZ).

The 56°N FZ was imaged with reflection seismic data in the subduction zone beneath the frontal prism^21^ (Fig. S1). Magnetic anomalies are offset ~85 km right laterally^20,22^ along 56°N FZ (Fig. 2). On the incoming oceanic plate, the 56°N FZ is located parallel to and along the southwest flank of the broad ridge topped by the Kodiak Bowie Seamount Chain (KBS; Fig. 1) and parallel to the Aja FZ. The epicenter and a ~70 km long linear band of westward trending aftershocks are located along the 56°N FZ. The main and aftershock focal mechanisms (Fig. 2) are dominated by strike-slip/oblique-slip with a few normal faulting events. Strike-slip motion prevails along N-S and E-W lineaments and the normal faulting aftershocks are aligned parallel to both the trench axis and the oceanic fabric.

**Figure 4:**
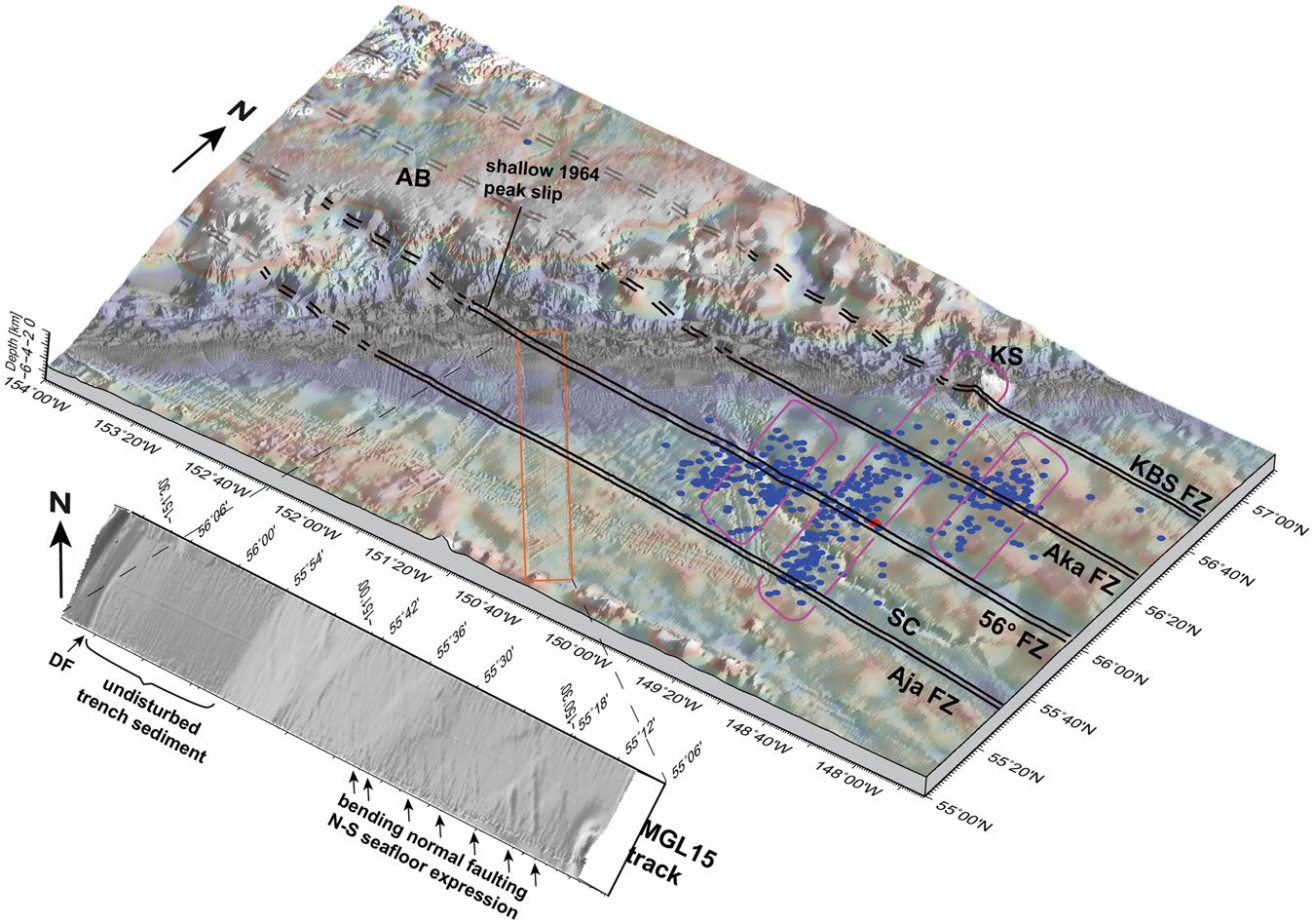
Perspective view of vertical gravity gradient (VGG; TOPEX^29^) overlain on bathymetric compilation. Bathymetry is illuminated to highlight E-W/N-S trending structures. Red dot marks main shock, blue dots M_W_ > 3.5 aftershocks (USGS/NEIC catalog^19^, https://earthquake.usgs.gov/earthquakes). Rupture along N-S trending magnetic anomalies are outlined in magenta. Double black lines mark FZs, dashed on upper plate (solid, where constrained by seismic images). Orange rectangle shows location of zoom on MGL15 bathymetric track, perspective view below. AB – Albatross Bank; DF – deformation front; SC – Surveyor Channel; KS – Kodiak Seamount; KBS – Kodiak Bowie Seamount Chain. Figure made with GMT vers. 4^50^.

Barriers to the limits of aftershocks are the Aja FZ in the south and the Kodiak Bowie Seamounts to the north (Figs 1 and 2). The first trend or linear alignment of aftershocks, parallels the N-S strike of magnetic anomaly 20 and 18 south of 56°N FZ^20^ (Fig. 2, inset). It extends from KS across Aka and 56°N FZs ~150 km around the centered main shock (Fig. 2). On either side of this central N-S trending aftershock lineament and both north and south of the 56°N FZ two sub-clusters are aligned with the adjacent magnetic anomalies^20^ (Fig. 2). Aftershocks cluster ~25 to 100 km seaward of and parallel to the trench axis where the oceanic plate is covered by the trench axis sediment fill. Modern seismic data, e.g. RV *Marcus G. Langseth* seismic line 15 (MGL15^6,23^), images broad areas of bend faults^6,24^ along the trench seaward flank (Fig. S1). The plate bending faults continue beneath undisturbed trench sediment at least to the Patton Murray seamount chain. High resolution multibeam tracks show the N-S trend of these normal bend faults^6,24^ instead of trench parallel fabric (Fig. 4). Besides these small-scale N-S trending bathymetric lineaments imaged in the high resolution multibeam patches, no linear N-S trend toward KS is obvious in bathymetric compilations^6,25–28^ (Figs 1 and 4). The E-W linear zones of the fracture zone system ruptured by the 2018 M_W_ 7.9 Gulf of Alaska earthquake correspond to subtle, long-wavelength minima, shallow and somewhat irregular furrows in GEBCO bathymetric data^27,28^ (Fig. 1), however these E-W trending zones may be clearly identified in the vertical gravity gradient (VGG^29^; Fig. 2). The surface expression of these lineaments terminates at the trench axis in the VGG, as well as the bathymetry (Figs 1 and 2).

## Discussion

The 2018 M_W_ 7.9 Gulf of Alaska earthquake occurred along two closely linked tectonic features (Fig. 2, inset): (1) right lateral offset N-S aligned ocean crustal fabric and (2) E-W oriented FZ system in the outer-rise of the Alaska subduction zone. The alignment of the first category aftershock cluster that parallels the N-S magnetic anomalies 20, 18 and 19 is similar to the N-S central Gulf of Alaska strike-slip 1987/88 earthquake sequence (Fig. 2). In contrast to the recent 2018 strike-slip event, the central Gulf of Alaska shear zone (GASZ^5^) aftershocks are aligned along one single lineament of oceanic fabric (magnetic anomalies 12 south of and 13 north of and slightly offset by 58° FZ^4,30^). The triggering farfield stresses of the 1987–1992 GASZ earthquake sequence, most likely, originate in the collision/subduction of the Yakutat Terrane^5^.

However, postseismic deformation model predictions for the 1964 great earthquake show maximum values in the Kodiak asperity^18^. Additionally, rupture along normal faults in the outer-rise region may be explained by a shallow rupture of the 1964 great Alaska earthquake, which then would have increased the Coulomb stress in the outer-rise region^31^. Therefore, we prefer a stress transfer from the subduction zone into the outer-rise region of the 2018 Gulf of Alaska earthquake sequence.

The 2018 Gulf of Alaska main shock and aftershocks activated the 56°N FZ system that formed during plate reorganization when the ancient Farallon spreading center encountered the North America Plate ~30 myr ago^32^. This required large adjustments of fractures to accommodate that change^20,33^. The downward flexure of the Pacific Plate into the Alaska Trench produces bending-related stresses in the lithospheric oceanic plate. To accommodate these stresses, the prevailing tectonic mechanism is an activation of pre-existing ocean-lithospheric structures rather than the creation of new faults resulting in outer-rise normal faulting (e.g.^34^). Plate bending stresses that are commonly accommodated by trench-parallel outer-rise extensional faulting are aligned N-S parallel to magnetic anomalies in the Alaska Trench outer-rise region, indicated by multichannel seismic (MCS) data^6,24^ and multibeam bathymetry^6,24^ (Fig. 4). At 30° obliquity to the trench axis, the crustal fabric commonly guides faulting^34,35^.

As unusual as this strike-slip intra-oceanic lithosphere earthquake was, it is not unique and has several features in common with two other tectonic settings: Sumatra (e.g.^15,16^) and Macquarie/Puysegur (e.g.^10,11,14^), that produced similar earthquake patterns. All three events (Sumatra, Macquarie/Puysegur and 2018 Gulf of Alaska) resulted in complex aftershock distributions following fault systems parallel and orthogonal to major FZs in the outer-rise region where extension usually occurs^14–16^.

**Figure 5:**
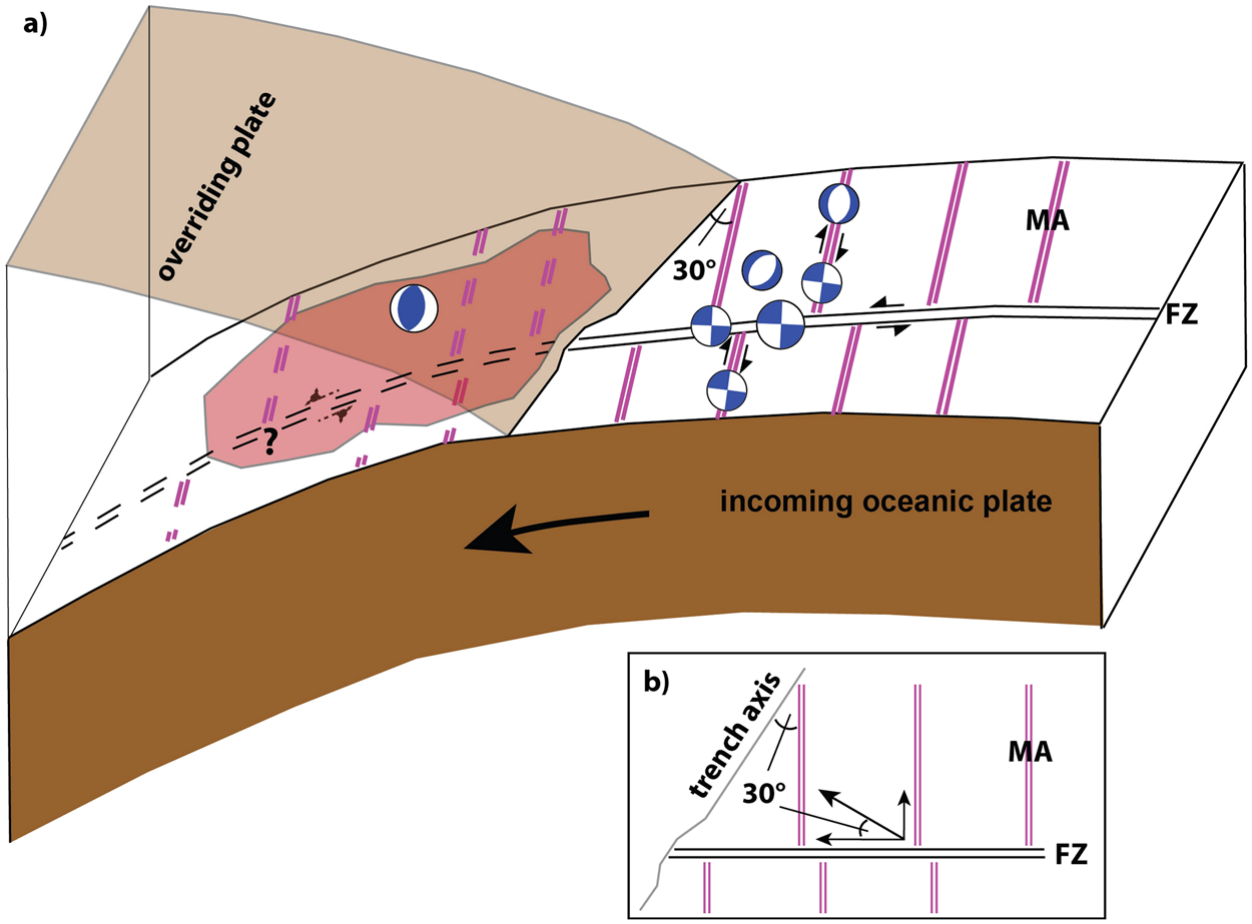
(**a**) Conceptual model of the main subduction zone characteristics where strike-slip earthquakes occur in the outer-rise region of the incoming oceanic lithosphere. Magnetic anomalies (magenta double lines) are offset by fracture zone (FZ) and trend ~30° oblique to the trench axis. Strike-slip reactivates pre-existing oceanic fabric and ruptures en échelon and orthogonal fault zones parallel to FZs, which offsets perpendicular magnetic anomalies. Red patch shows interplate rupture zone with shallow slip that extends almost to the trench. Earthquake doublets occur and each of them may trigger the other mechanism. Slip along FZs may occur downslope of the trench, as indicated by dashed strike-slip arrows. (**b**) Schematic sketch of strain partitioning forces to re-activate preexisting ocean lithospheric structures: Trench axis normal plate convergence vector is divided into an ocean fabric and a FZ fraction leading to strike-slip fault rupture which dominates extensional faulting in the outer-rise region. MA – magnetic anomaly; FZ – fracture zone.

For both Sumatra and Macquarie/Puysegur, plate convergence is oblique, resulting in an oblique alignment of ocean-lithosphere magnetic anomalies that are obliquely aligned (~30–35°) to the trench axis. Proposed strain partitioning that accommodates oblique plate convergence^36^ results in a dual rupture mode of interplate thrust events and interplate (Macquarie/Puysegur^11^) or upper plate (Sumatra^15^) strike-slip events. In contrast, plate convergence along this portion of the Alaskan subduction zone is trench-normal with inherited ocean-lithospheric magnetic anomalies at ~30–35° to the trench axis as well. Here, we expand the strain partitioning model^36^ for more general and complex cases to accommodate the oblique alignment of inherited ocean-crustal fabric and FZs instead of oblique plate convergence. We propose that a significant condition promoting large orthogonal intra-oceanic strike-slip earthquakes near subduction zones may be the oblique alignment of FZ systems and crustal fabric ~30–35° to the trench axis (Fig. 5).

Paleogene oceanic crust is generally considered to be without substantial stress (e.g.^37^). Thus, proximity to a subduction zone may be significant to understanding oceanic intraplate earthquakes in Paleogene crust. A driving mechanism to produce major earthquake slip in intra-oceanic lithosphere may be stress transfer from the subduction zone. This could either be due to stress release along the subduction megathrust after a great earthquake, or stress accumulation along the subduction megathrust late in the seismic cycle prior to a great subduction earthquake. The result could be an earthquake doublet involving an interplate thrust event and an intra-oceanic plate strike-slip event, described by two triggering mechanism models^8^: In the first model the initial great rupture occurred along the plate interface and triggered the intraplate strike-slip event, whereas in the second model, the triggering interaction occurred in reverse order. The first triggering mechanism occurred after the 2004 Sumatra great megathrust earthquake which initiated increased (strike-slip) seismicity in the adjacent oceanic lithosphere^16^. Due to the high levels of interspersed intra- and interplate seismicity in the Macquarie/Puysegur region (e.g.^11,14^) changing triggering interaction between intra- and interplate events is inferred, hence the combination of the two above proposed triggering mechanisms^8^. The 2018 Gulf of Alaska earthquake might have occurred either as a delayed postseismic response to the 1964 great earthquake or during the interseismic period as a result of stress accumulation and strain release from compressional convergent forces and locking of the adjacent subduction megathrust. We favor stress transfer from the subduction zone as the driving mechanism for the current main and aftershock sequence since the orientation of moment-tensors is left-lateral in the E-W direction (Fig. 2, inset) at present, in contrast to the right-lateral offset of magnetic anomalies representing the historic stress regime during FZ system formation.

The 2018 Gulf of Alaska events involved slip on two zones of weakness in the oceanic lithosphere. The main M_W_ 7.9 strike-slip event was followed by outer-rise aftershocks where normal fault events are common. The strain release from the coseismic strike-slip event may have transferred stresses into the outer-rise domain and there generated normal bend faulting as well as normal faulting events parallel to magnetic anomalies. However, the relation to great subduction thrust earthquakes is not clear, because the increase in ocean-lithospheric plate seismicity occurred 54 years after the great megathrust earthquake and not promptly thereafter as in the Sumatra earthquakes^16^. Perhaps locking of the subduction zone required a long period of plate interface processes.

Weak seismicity (mostly M_W_ < 4) extends locally into the subduction system at the prolongation of the Kodiak Bowie Seamount chain (Fig. 1). Rupture of the Investigator FZ down into the subduction zone beneath Western Sumatra was shown with a dense local offshore seismic network^38^. An offshore seismological network along the Alaska margin would have shed more light on rupture mechanisms and accurate (depth-) relocation of the weak seismicity observed in the upper plate. A local seismic network might record the weak seismicity from rupture of the FZ system (intraplate in the subducting slab) beneath the upper plate or alternatively from induced seismicity along the subduction thrust (interplate) or stress transfer into the upper plate.

The 2018 Gulf of Alaska earthquake is a third recorded, global, large intra-oceanic plate strike-slip event. It is likely that historic events with the same mechanisms have occurred before instrumental seismology. Also, this probably is not the last major or great strike-slip event. Other active margins satisfy the requirements of having magnetic anomalies trending ~30° oblique to the trench axis: i.e. Aleutian, Ecuador, Guatemala, Kuril, Solomon (Woodlark), with some that produced great megathrust earthquakes in the shallow part of the subduction zone^8^ (and references within).

## Conclusion

The 23 January 2018 M_W_ 7.9 strike-slip oceanic lithosphere earthquake is a rare example of an oceanic intraplate strike-slip rupture. Its occurrence was unexpected because the structures involved are covered by thick turbidites that smoothed the underlying seafloor bathymetry. Despite few ship survey data, the combination of several regional geophysical observational datasets gives insight into the complex tectonic setting/history of this earthquake and its aftershock distribution.

Given the global history of such unusual strike-slip intra-oceanic plate earthquakes, one common feature is proximity to a subduction zone that produces great earthquakes with rupture close to the trench. All three rare events, Macquarie/Puysegur, Sumatra and Gulf of Alaska, resulted in complex en échelon and orthogonal fault rupture of pre-existing ocean plate fabric and FZ systems (Macquarie/Puysegur and Sumatra^8^ and references therein). Instead of oblique convergence, a key tectonic feature that leads to strain partitioning for such events is the trend of the ocean plate fabric at ~30–35° to the trench axis and orthogonal FZs. Similar scenarios may occur in other subduction systems that ruptured in great megathrust earthquakes where oceanic fabric on the incoming oceanic lithosphere trends ~30–35° obliquely to the trench axis (e.g. Aleutian, Guatemala, Kamchatka, Kuril, Solomon/Woodlark).

## Methods

### Bathymetry compilation

For most of the figures we used the GEBCO 2014 bathymetry grid^27,28^. Figure 4 includes a bathymetric compilation using high resolution multibeam data^6,25,26^, where available, set into the GEBCO 2014 grid^27,28^. Multibeam data were cleaned and processed using MB-System^39,40^.

### Multichannel seismic data

USGS legacy multichannel reflection seismic (MCS) lines acquired with USGS vessel RV *S.P. Lee* in 1977 and 1981 (cruises L-7-77-WG available at, https://walrus.wr.usgs.gov/namss/survey/l-7-77-wg/ and L-7-81-WG available at, https://walrus.wr.usgs.gov/namss/survey/l-7-81-wg/) and a recently acquired line with RV *Marcus G. Langseth* (line MGL15^23^ of cruise L-09-11-GA, available at, https://walrus.wr.usgs.gov/namss/survey/l-09-11-ga-mcs/) were processed. The processing procedure involved frequency filtering, multiple suppression and trace interpolation for the legacy data. MCS data were sorted for common image gathers, which were used for iterative semblance velocity model-building. Velocity model-smoothing was applied to reduce small scale artefacts. Pre-stack depth migration with the final velocity model and stacking provided the subsurface image.

### Earthquake back-projection

We implemented a time-domain back-projection^41^, building on a large body of earlier work (e.g.^42,43^) to evaluate the high-frequency emissions of the 23 January 2018 Mw 7.9 strike-slip Gulf of Alaska Earthquake. The back-projection method analyzes the coherence of multiple signals arriving at a seismic array within narrow windows during the rupture of a larger event, assuming as sources candidate locations on a (generally) 2D grid around the hypocenter. For each potential source point the theoretical arrival times to each receiver of the array are calculated based on a 1D velocity model (here IASP91^44^). The signals within a narrow sliding window of length L are aligned according to these calculated travel times and finally summed to form a beam trace. Finally, the source point with the largest total beam power in each time window is selected as the most likely emission point for seismic waves for this time window. We additionally carry out a semblance (S) analysis^45,46^, which measures the coherence of waveforms and is less likely to be dominated by the most energetic part of the rupture but loses information on the relative importance of various rupture phases (S = 1 corresponds to a perfect coherence).

After removal of traces with poor signal-to-noise ratio, our dataset consists of 254 vertical velocity component seismograms from stations in central and eastern North-America and a few from the Caribbean (most stations at distances 35–53°) and 176 stations from Europe (most stations between 65° and 89°). Back-projection data were provided by the Data Management Center (DMC) of the Incorporated Research Institutions for Seismology (IRIS)^47^. A zero-phase fourth order Butterworth filter with a pass band between 0.5 and 2.0 Hz was applied to the data^48^. The back-projection assumes a source grid with a spacing of 4 km around the hypocenter area at a constant depth of 25 km, a time window of L = 6 s with time steps of 0.5 s. Both arrays were back-projected separately and the maps of beam energy or semblance were obtained by multiplying such independent back-projections results in space (2D grid) and time for our final results (Fig. 3).

To take into account the 3D Earth heterogeneity and its effects on the arrival times and consequently, on the constructive interference necessary to illuminate the rupture, the back-projection method includes static station corrections. The station statics supply station-specific time-shifts relative to the theoretical arrival times to maximize the correlation between the waveforms registered at the array. In order to determine these statics a pilot time window with known event parameters is needed. Here, we calculated the time-shift values based on the hypocenter using the adaptive stacking process^49^. Based on this method, the north-central America data set was aligned using the first 6 s after the theoretical P-phase arrival (filtered between 0.5–2.0 Hz). As the European data were at a larger distance and thus noisier, a wider window of 8.5 s had to be used (filtered between 0.5–2.0 Hz). The coherence of the selected waveforms and the time-shift values estimated are shown in Figure S3.

Finally, part of the North America waveforms were affected by polarity flips as the array is bisected by the nodal plane (Fig. S4). Therefore, we flipped the down polarity to up before the time-shift estimation for the arrival times and the back-projection. The procedure considered two steps, first the waveforms were filtered between 0.01 and 2.0 Hz and an automatic method detected the down and up polarity based on the first pulse that exceed by a factor of 4 the positive or negative median of a 60 s window ending five seconds before the theoretical arrival time. In order to identify waveforms near the nodal plane with ambiguous polarity, the time windows between 4 and 7 s after the arrival time for signals recognized with a down polarity were stacked and correlated with waveforms identified as up polarity near the nodal plane and above the transect presented in Fig. S5. Signals with a correlation coefficient higher than 0.3 were considered to be ambiguous and removed from the final analysis. The remaining down pulses identified during the first stage were flipped to up for the back-projection analysis^29^.

## Electronic supplementary material

Supplementary Information

Supplementary MovieS1
