# Supplementary material for: Strike-slip 23 January 2018 MW 7.9 Gulf of Alaska rare intraplate earthquake: Complex rupture of a fracture zone system
Source: Sci Rep. 2018 Sep 12;8:13706. doi: 10.1038/s41598-018-32071-4 (PMC6135818; doi:10.1038/s41598-018-32071-4)
Supplement: Supplementary file 1 — Supplementary Information [file 41598_2018_32071_MOESM1_ESM.pdf]

Strike-slip 23 January 2018 M_W 7.9 Gulf of Alaska rare intraplate earthquake: Complex rupture of a fracture zone system

Anne Krabbenhoeft* [1], Roland von Huene[2], John J. Miller[3], Dietrich Lange[1], and Felipe Vera[4, 5]

**Corresponding author, email: akrabbenoeft@geomar.de.*

[1]*GEOMAR Helmholtz Center for Ocean Research Kiel, Wischhofstr. 1-3, 24148 Kiel, Germany.*

[2]U.S. Geological Survey*, Scientist Emeritus, 800 Blossom Hill Road, Los Gatos, CA 95032, rhuene@mindspring.com.*

[3]*U.S. Geological Survey, Scientist Emeritus, Denver Federal Center, Denver, CO 80225, jmiller@usgs.gov.*

[4]*Helmholtz-Zentrum Potsdam, Deutsches GeoForschungsZentrum GFZ, Telegrafenberg 1, 14473 Potsdam, Germany, fvera@gfz-potsdam.de.*

[5]*Freie Universität Berlin, Malteserstr. 74–100, 12249 Berlin, Germany.*

SUPPLEMENTARY

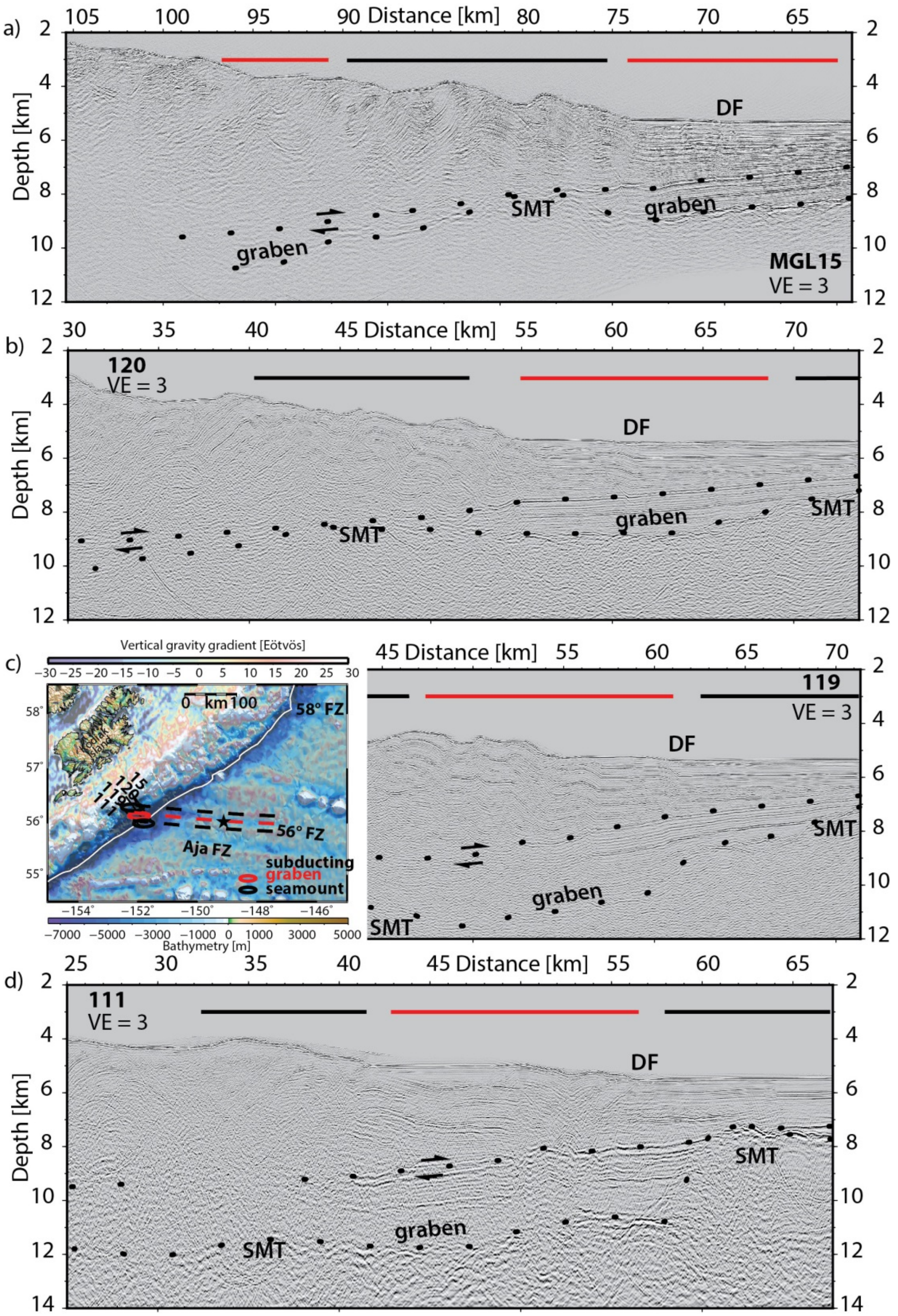

Figure S1, a) thru d): Depth-migrated seismic MCS-data showing the marine forearc structure including the subducted oceanic plate 56°N FZ. Locations of the trench-perpendicular seismic profiles are shown in the inset c) and are oriented northwest to southeast. Dotted lines highlight plate interface and oceanic basement; black/red lines mark extent of horsts/grabens on MCS images. Seamounts or horsts and grabens of the subducting 56°N FZ are imaged. DF – deformation front. **Inset c)**: VGG (TOPEX[29]) superpositioned on bathymetry (GEBCO 2014[28]). Star marks the 23.01.2018 M_W 7.9 Alaska earthquake.

Dashed lines follow subducting FZ system. Red marks negative relief (grabens) and black marks positive relief (horsts). Ovals mark continuation of subducting 56°N FZ system imaged in seismic profiles. Figure made with Seismic Un*x, Release No. 44[54] and GMT vers. 4[50].

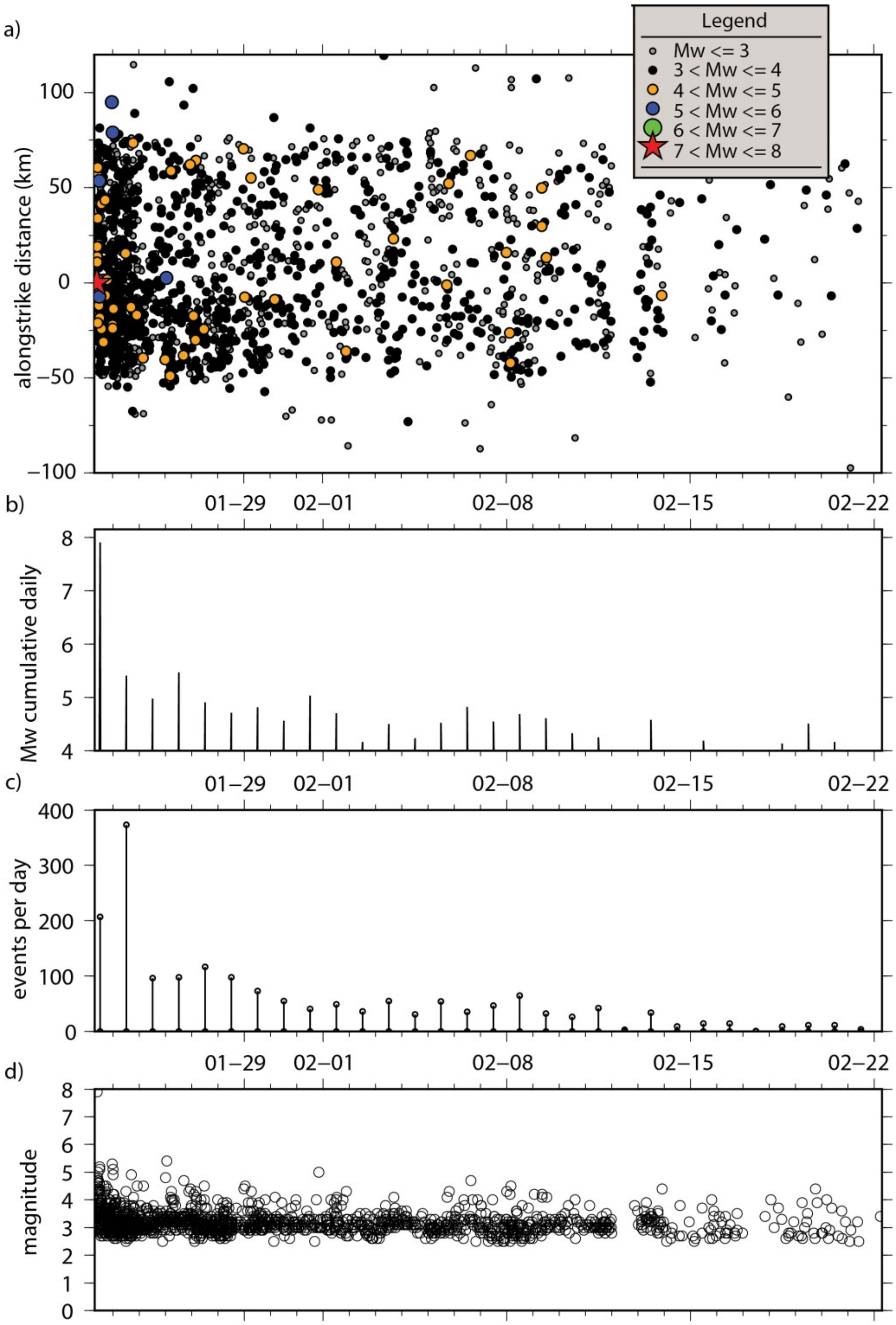

Figure S2: a) Aftershocks of the M_W 7.9 2018 Alaska event plotted along-strike direction (centered at the main shock), against time. The main shock is indicated by red star. **b)** Cumulative magnitude vs. time. **c)** Events per day vs. time. **d)** Event magnitude vs. time. Figure made with GMT vers. 4[50].

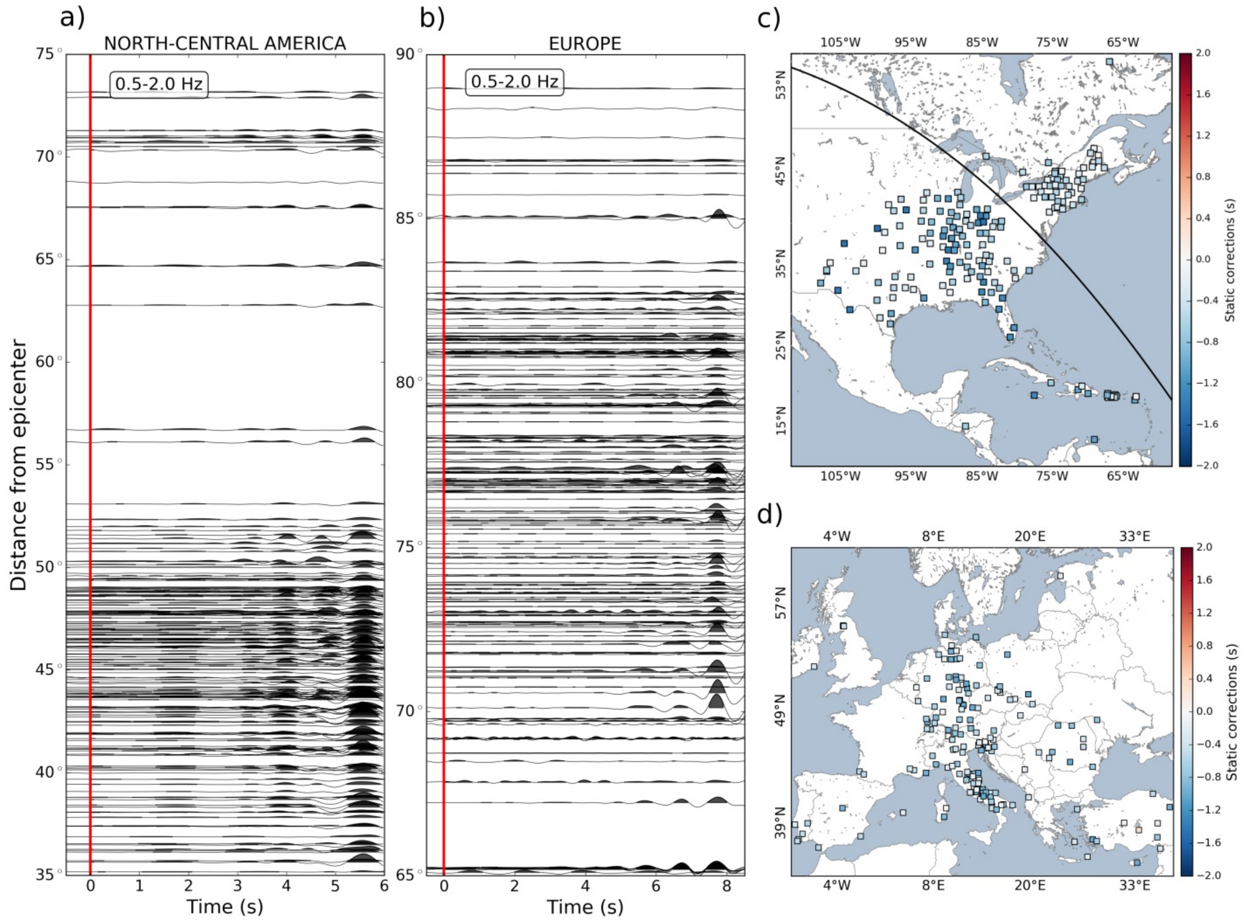

Figure S3: Waveforms and station corrections. **a)** North-American waveforms (0.5 – 2.0 Hz) and **b)** European waveforms (0.5 – 2.0 Hz) with the rupture onset aligned according to the predicted arrival time plus station correction. The vertical red line shows the theoretical P-phase arrival time based on the IASP91 model[44] and the **c)** and **d)** present the stations corrections for North-America and Europe. The black curve in **c)** shows the focal mechanism nodal plane projection through the North-America array. Figure made with Obspy[52] & matplotlib[53].

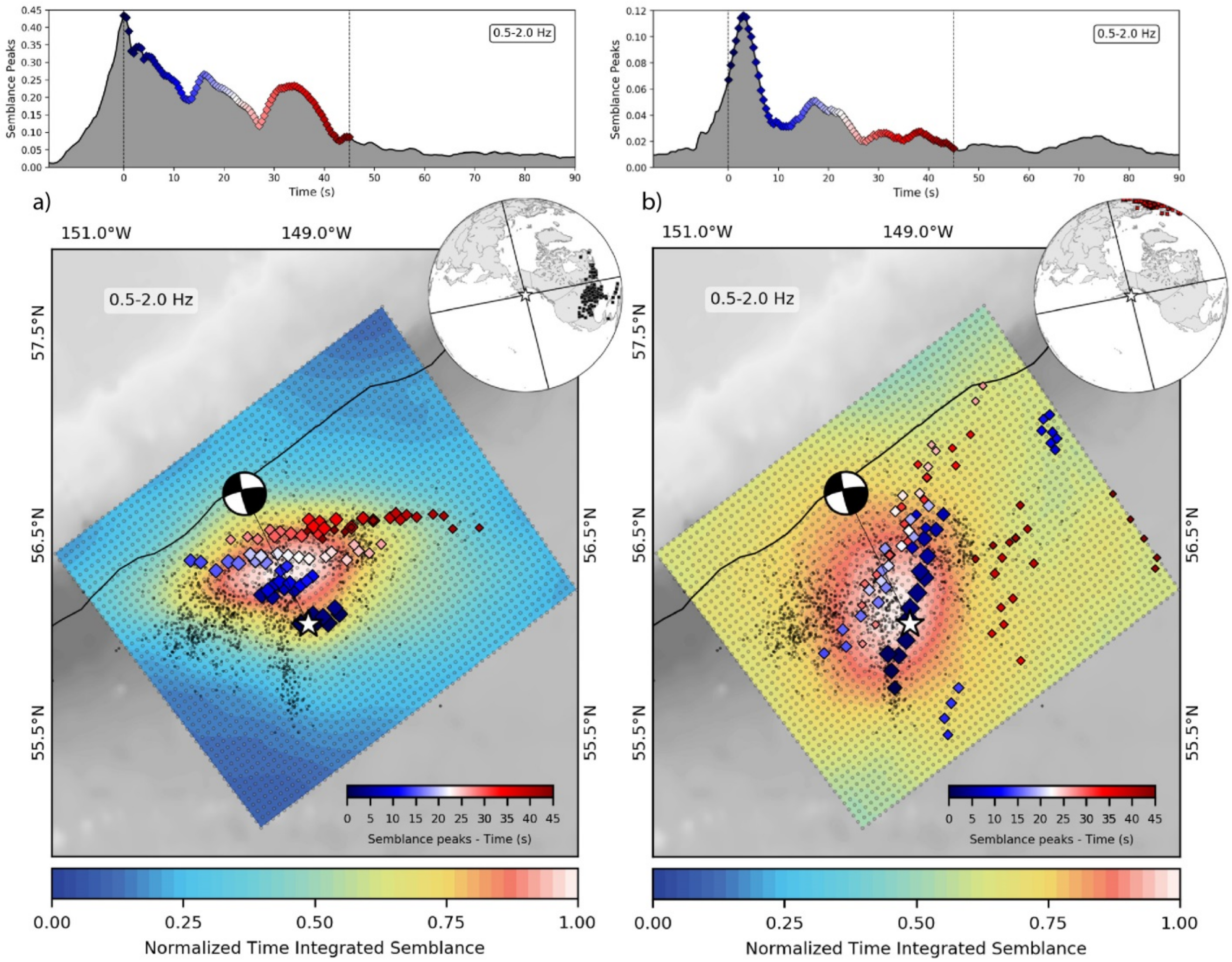

Figure S4. Top: Time history of semblance for each time window of L= 6 s centered at t=0. Normalized time integrated semblance (color image) and semblance peaks (diamonds) for the 23 January 2018 Mw 7.9 strike-slip Gulf of Alaska Earthquake back-projection (0.5 – 2.0 Hz). **a)** Back-projections results using the North America stations and **b)** the European stations. The semblance peaks are denoted by colored diamonds related to the first 45 s of the rupture time. Other elements are as in Fig. 3. In interpreting these figures it has to be noted that apparent streaks in the dominant array direction (here, North America and Europe, approximately to the E and NNE, respectively, often arise from the so-called swimming artifact). By combining both arrays, this type of artifact is suppressed (Fig. 3, main text). Figure made with Obspy[52] & matplotlib[53].

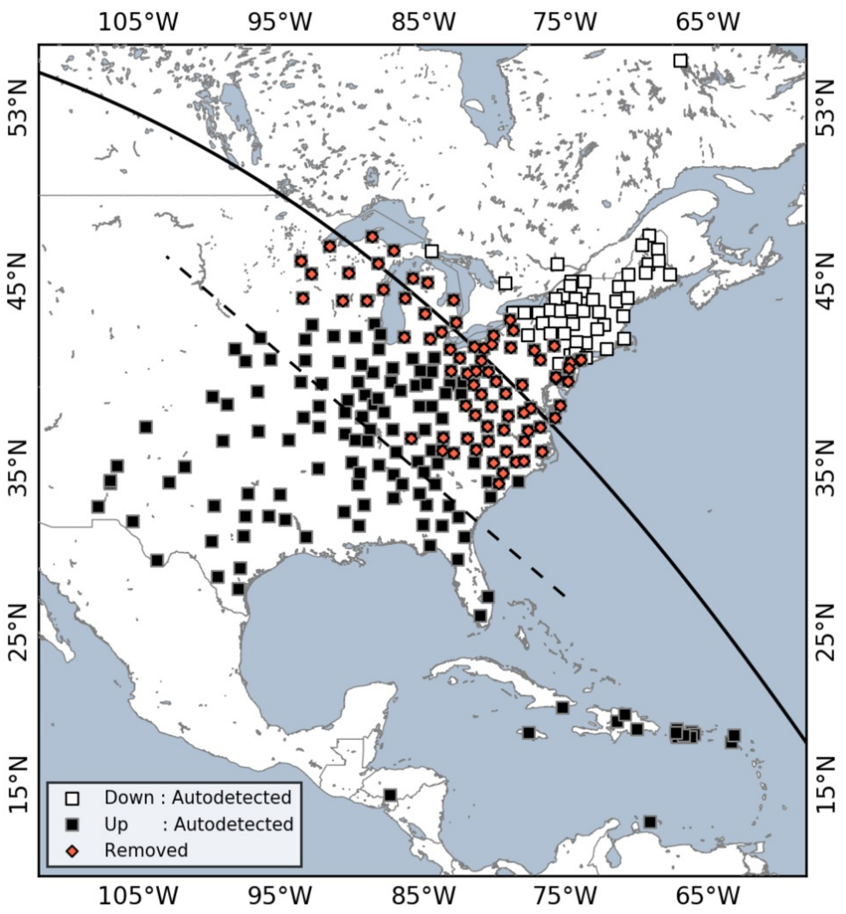

Figure S5: Polarity evaluation in north-central America waveforms. The black curve tracks the focal mechanism nodal plane projection. An automatic method detected the down (white squares) and up (black squares) polarity based on the first pulse that exceed by a factor of 4 the positive or negative median of a 60 s window ending five seconds before the theoretical arrival time. The down pulses were subsequently flipped to up for the time-shift corrections and back-projection analysis. The orange diamonds indicate the stations removed (76 in total) during a second stage, which were recognized initially as up pulses. This second stage involve a cross correlation (CC) between the stack based on the down pulses previously identified and the remaining up pulses located above the segmented black transect. The signals with a correlation coefficient higher than 0.3 were considered to be ambiguous and were removed. Figure made with matplotlib[53].

Movie S1: Temporal rupture normalized energy (colored) and semblance (marked by X) peak propagation of the 23 January 2018 Gulf of Alaska unusual strike-slip oceanic lithosphere earthquake. Movie made with Obspy[52] & matplotlib[53].
